# Diffuse Xanthogranulomatous pyelonephritis: A Rare Disease With A Common Presentation

**DOI:** 10.7759/cureus.44118

**Published:** 2023-08-25

**Authors:** Ahmed Gamal Sayed, Tafe Badghaish, Nivan Abdo, Abdallah Nasir

**Affiliations:** 1 College of Medicine, Alfaisal University, Riyadh, SAU; 2 Pediatrics, Maternity and Children Hospital Makkah, Makkah, SAU

**Keywords:** fever of unknown origin, pediatric surgery, case report, nephrectomy, pyelonephritis, xanthogranuloma

## Abstract

Xanthogranulomatous pyelonephritis (XGP) is a distinct entity characterized by chronic granulomatous changes in the renal parenchyma associated with renal destruction and urinary tract abnormalities, most often from obstruction or infection in the urinary tract. We have presented the case report of a girl with fever, abdominal pain, vomiting, anorexia, and weight loss. Computed tomography of the abdomen showed multiloculated cystic lesions with calcifications and a psoas muscle abscess, which tested positive for Escherichia (E.) coli. Histopathology revealed lipid-laden macrophages, multinucleated giant cells, and fibrosis. Nephrectomy and drainage of the psoas abscess were done. XGP, although rare, should not be confused with renal tumors and should be considered in children presenting with fever and urinary tract symptoms. Early diagnosis can be reached with CT. Nephrectomy is the definitive treatment.

## Introduction

Xanthogranulomatous pyelonephritis (XGP) is a disease process in which there is a chronic, severe, suppurative, and inflammatory process that results in the destruction and replacement of the renal parenchyma by granulomatous tissue containing histocytes and foamy cells [1]. The disease is classified according to its extent. In focal or segmental XGP, the disease remains within the renal cortex, whilst in diffuse XGP, it extends beyond the cortex with pelvic communication. Although rare, XGP can have fatal complications, including perinephric, psoas abscess, nephron-cutaneous fistula, and reno-colic fistula [2]. The first case of XGP was reported in 1916 by Schlag-enhaufer [3]. The term Xanthogranulomatous pyelonephritis was used in 1944 by Osterlind [1]. XGP, and its ambiguous clinical appearance, may mimic renal tuberculosis or neoplastic tumors, including renal cell carcinoma, resulting in a challenging diagnosis preoperatively [4], but now, computed tomography has allowed the determination of suspected cases of this otherwise rare condition. A confirmed diagnosis is, nevertheless, based on the histopathological examination of the specimens [5]. We reported a rare manifestation of XGP in a girl who presented with prolonged fever, abdominal pain, and weight loss without an apparent abdominal mass. Abdominal mass was the presenting symptom in 39.4% of a cohort study [6] and the most common finding in a physical examination in another study [7].

## Case presentation

A 13-year-old female presented with a history of a 40-day fever, abdominal pain for one month, and three days of limping before presentation. Initially, the fever was low-grade and responded to paracetamol; she was diagnosed with tonsilitis and was given amoxicillin at the health center without improvement. A week after the fever, she developed mild right-sided abdominal pain, relieved by applying pressure, such as lying down ipsilaterally, and aggravated by lying on the left side. Gradually, the pain worsened, as it progressed to the inguinal region and intensified while walking, which caused her to lean forward to minimize the pain. Additionally, she complained of vomiting after meals, especially in the morning, for two weeks before admission, which led to significant weight loss. She had no history of urinary tract infections, no history of preceding trauma, renal stones, or congenital anomalies; her family history was also unremarkable. Her temperature was 38.5 °C, pulse 139 bpm, respiration 25/min, blood pressure 87/54 mmHg, height 144 cm in the 10th percentile, and weight 30 kg in the 5th percentile. Physical examination revealed an alert and cooperative child with a fever. She had mild pallor, and there was right abdominal tenderness with no palpable masses. The hip was in a flexed position while another systemic exam was unremarkable. Laboratory investigations are illustrated in Table 1.

**Table 1:** Laboratory values Hct: hematocrit; MCV: mean corpuscular volume; MCH: mean corpuscular hemoglobin; MCHC: mean corpuscular hemoglobin concentration; ESR: erythrocyte sedimentation rate; CRP: c-reactive protein

Variable	Result	Reference Range
WBC	17.25×10^3^/UI	4-13 ×10^3^/UI
Neutrophils	12.01×10^3^/UI	4-13×10^3^/UI
Lymphocytes	3.4×10^3^/UI	1-4×10^3^/UI
Monocytes	1.68×10^3^/UI	0.1-1×10^3^/UI
Eosinophils	0.09×10^3^/UI	0.1-1×10^3^/UI
Basophils	0.07×10^3^/UI	0.02-1×10^3^/UI
RBC	4.35×10^3^/U	3.9-4.6×10^3^
Hb	9.7 mg/L	11-16 mg/L
Hct	34.4	35-45
MCV	79.1 FI	77-93 FI
MCH	24.6	25-33
MCHC	31.1 g/dL	31-36 g/dL
Platelets	576/UI	155-345/UI
ESR first hour	112mm/hr	1-20mm/hr
CRP Latex	positive	-
CRP	25.27 mg/dL	0-0.5 md/dL
Creatinine	36 umol/L	44-88 umol/L
Sodium	134 mmol/L	138-145 mmol/L
Chloride	95.6 mmol/L	98-107 mmol/L
Potassium	4.41 mmol/L	3.5-5.1 mmol/L

**Figure 1:**
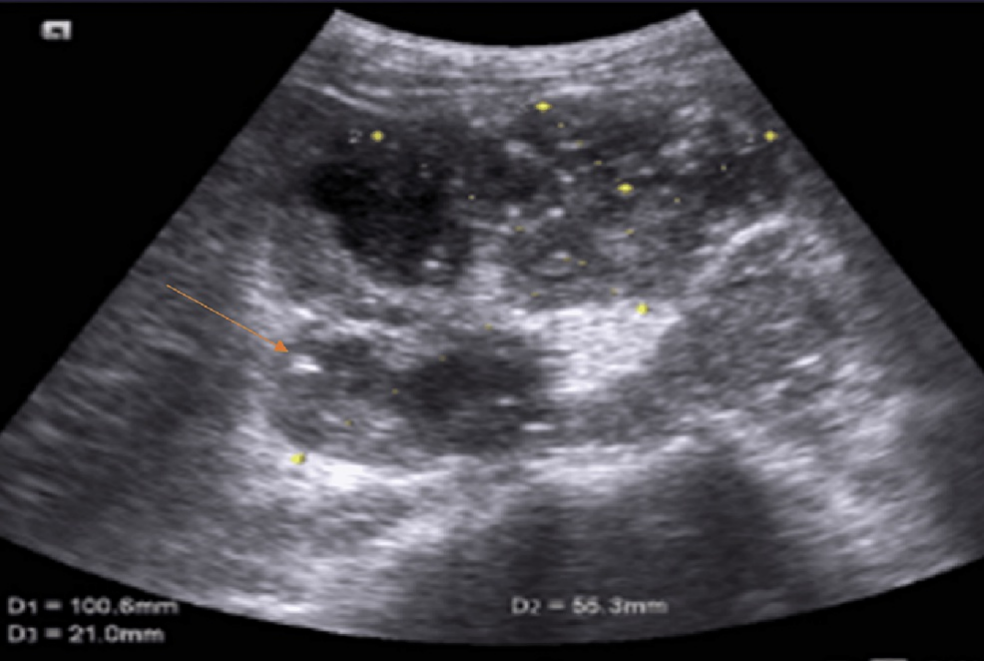
Renal ultrasound

Other renal and hepatic profile parameters, along with the urinalysis, were all normal. There was no hematuria, proteinuria, and no casts. The blood culture taken during the fever was negative and urine cultures were also negative. Furthermore, investigations for other causes of fever of unknown origin were negative such as malaria, typhoid, tuberculosis, hepatitis, CMV, and brucellosis. An abdominal ultrasound was done for the abdominal pain and revealed large hydronephrotic shadows with multiple echogenic particles indicative of pyelonephritis (Figure 1).

**Figure 2:**
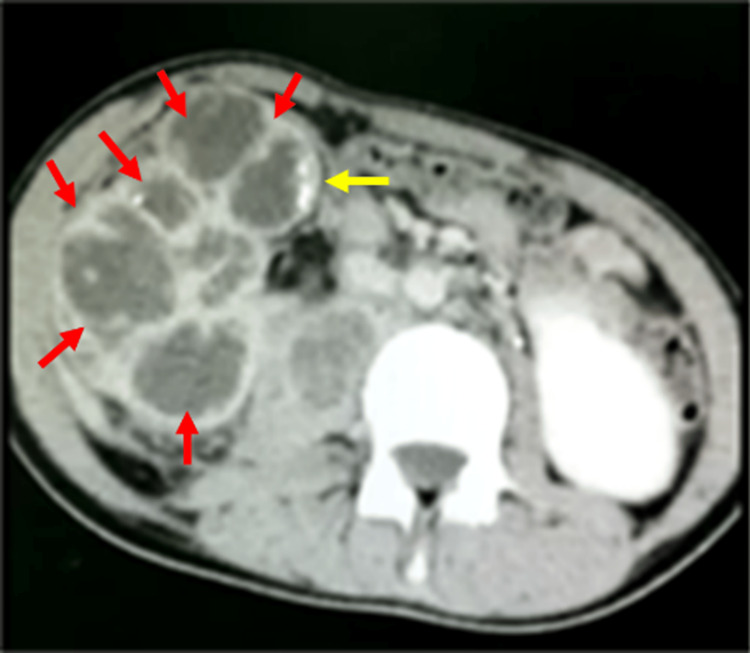
Computed tomography of the right kidney Red arrows: Kidney cysts; Yellow arrows: Calcifications

Computed tomography (CT) abdomen showed multiloculated cystic lesions with peripheral enhancement occupying the right kidney and calcifications scattered all over the kidney (Figure 2). The psoas muscle had multiple non-communicating regions with peripheral enhancement.

**Figure 3:**
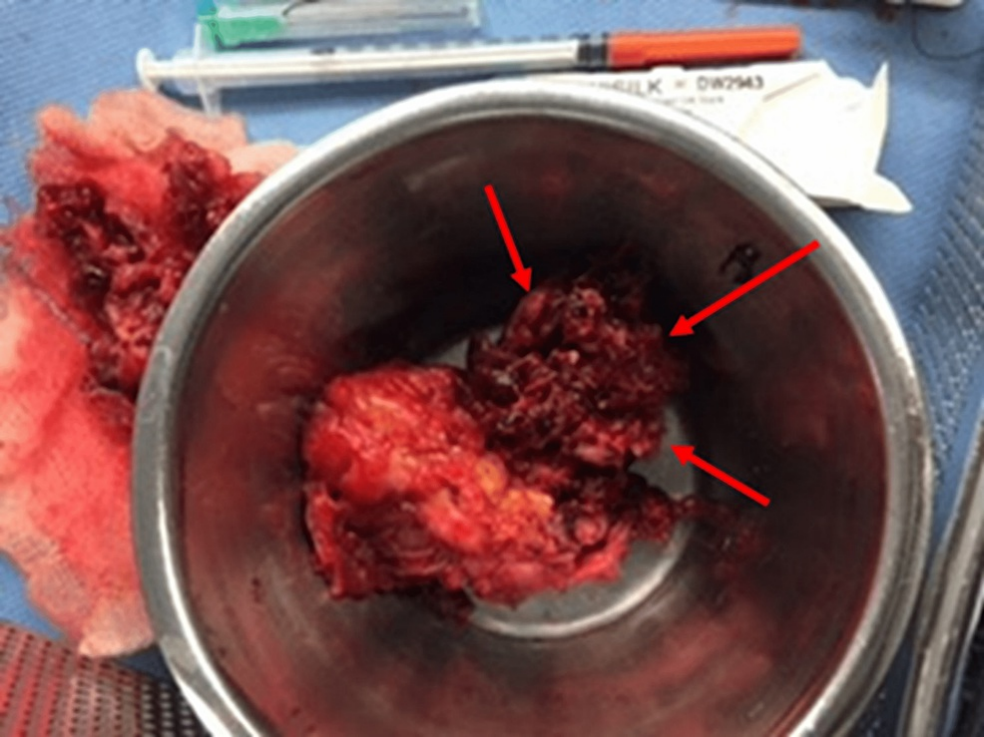
Gross appearance of the right kidney post-operative Red arrows: The atrophied segment of the affected kidney

The patient was started on ceftriaxone, but the fever did not respond. There was delayed excretion (up to four hours) in the intravenous contrast urography. Consequently, the patient was diagnosed with XGP and underwent percutaneous nephrostomy tube placement, which drained thick pus. Fluid aspirated from the right kidney showed a positive culture for Escherichia coli, which was sensitive to ceftriaxone, gentamycin, and ciprofloxacin, and polymerase chain reaction (PCR) for tuberculosis was negative. Gentamycin was added to the treatment plan along with ceftriaxone. Her fever continued to spike, so a right nephrectomy with psoas abscess drainage was done, following which her condition improved dramatically, and the fever resolved. The kidney appeared atrophied with a distorted shape (Figure 3).

**Figure 4:**
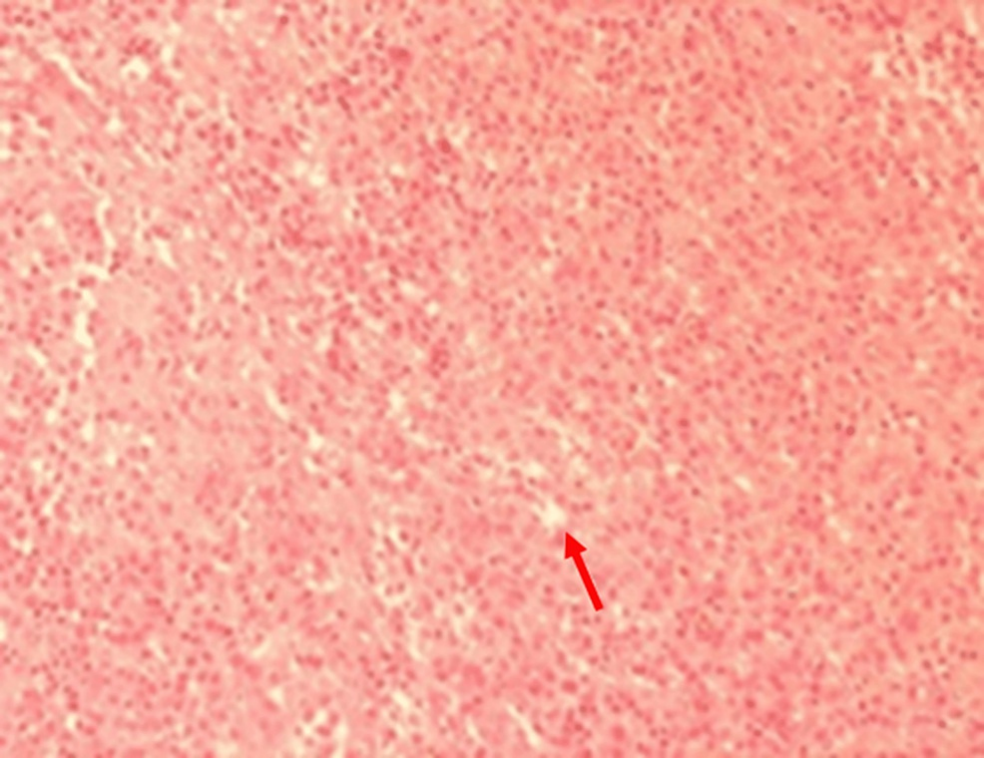
A histopathology section with Sudan III stain under 10× magnification Red arrow: Foamy macrophages

Histopathology of the kidney showed nodular replacement of the renal parenchyma exhibiting a zonal pattern and central nidus of necrotic debris and neutrophils (microabscesses) with a mixture of inflammatory cells, including lymphocytes and plasma cells. The nidus was surrounded by sheets of lipid-laden macrophages with abundant clear cytoplasm (Figure 4), spindle-shaped fibroblasts, and multinucleated giant cells. The residual glomeruli were sclerotic, and the parenchymal arteries thickened.

**Figure 5:**
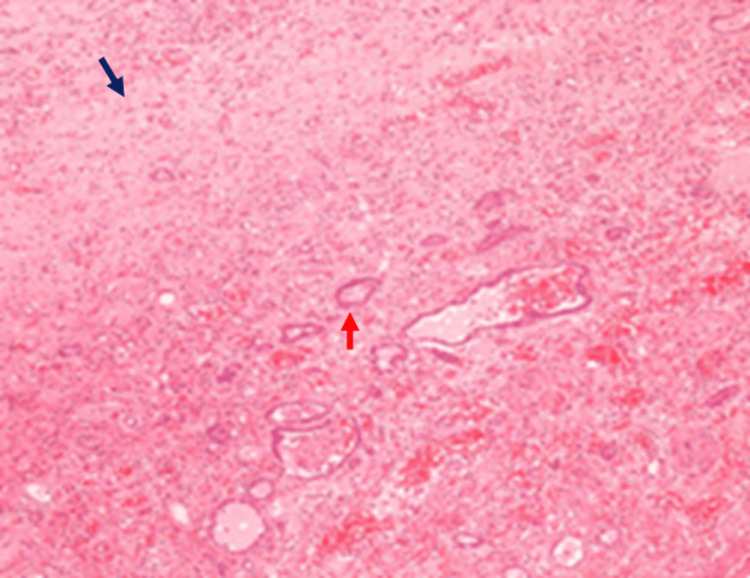
A histopathology section under hematoxylin and eosin stain with 10× magnification Black arrow: Fibrosis; Red arrow: Hyalination and scarring

In such cases, the tubules are often dilated. There was extensive fibrosis, hyalinization, congestion, focal calcifications, and lymphoid aggregations, besides fragments of fatty tissue exhibiting typical chronic inflammatory cell infiltration (Figure 5). No ureter or renal vessels could be identified.

 The patient had a smooth postoperative course and was doing well on further follow-up for one year in the outpatient clinic.

## Discussion

XGP is a rare disease occurring in children, which accounts for six out of 1000 surgically confirmed cases of chronic pyelonephritis [8]. More common in boys and usually affecting children <8 years of age [9], the disease has been reported as early as in a two-month-old infant [8]. There have been 200 reported cases until now [1]. The most reported symptoms of XGP are fever, abdominal pain, anorexia, and weight loss; a palpable flank mass is the most frequent finding on physical examination. Lower urinary tract problems and gross hematuria comprise other standard features [10]. Our patient developed a prolonged fever with abdominal pain and vomiting, which worsened without the presence of an abdominal mass. The diffuse type is more common in children than segmental or focal forms.

The etiology of XGP is mainly unknown, but nearly two-thirds of the time, it is accompanied by kidney stones and concurrent infections with anaerobic organisms such as Proteus mirabilis, Klebsiella spp., Staphylococcus aureus, Enterococcus spp., Pseudomonas spp., and Streptococcus spp. [4]. Prolonged ureteric obstruction due to renal stones (more so with staghorn variety) is observed, but not confirmatory, in 80% of patients with XGP [9]. Additional predisposing factors include chronic ureteropelvic junction syndrome, ureteropelvic duplication, tumors of the urinary bladder, and chronic interstitial nephritis, besides certain comorbid conditions such as chronic viral hepatitis C, rheumatoid arthritis, diabetes mellitus, cirrhosis, and obesity [10]. Therefore, XGP should be considered a differential diagnosis in young children presenting with symptoms of an abscess (perirenal or in the psoas), renal mass, or non-functioning kidney with or without chronic renal calculus disease [7].

The differential diagnoses of XGP include pyelonephritis, tuberculosis, perinephric abscess, and renal cell carcinoma [10]. Additional laboratory findings from a differential blood count analysis, such as leukocytosis and anemia [9], offer significant evidence of an underlying infection or inflammation; our patient, too, had leukocytosis with raised ESR. Furthermore, abnormalities in liver function are found in about 50% of XGP patients [9], and urinalysis typically reveals leukocytes, bacteriuria, and proteinuria. In such cases, urine culture and sensitivity analysis are essential to ascertain the offending organism and administer antibiotics accordingly [9]. Radiological imaging with a CT scan allows excellent visualization of the abdomen [11], which often reveals the presence of focal or diffuse enlargement of the involved kidney, the typical 'bear paw' sign - thinned out cortices and dilated calyces with thick walls, and the presence of a large staghorn calculus in the affected renal pelvis. Additionally, typical inflammatory changes may be observed along the fascial planes, spreading throughout the retroperitoneum encompassing the adjacent organs - pancreas, colon, and abdominal wall. In a case series on XGP published in 2011, the preoperative diagnostic rates ranged from 22% to 46.2% [10]. Histopathological examination of the resected mass usually shows a complete replacement of the renal parenchyma with lipid-laden (foamy) macrophages [12]. Antibiotics have only a secondary role in the treatment of XGP, with the mainstay treatment being a nephrectomy [1]. Following nephrectomy, the prognosis is usually good when the affected kidney is removed. A complete nephrectomy may not always be necessary; however, Deng et al reported a case of XGP in which a child with non-responsive fever underwent a partial nephrectomy, and showed complete subsidence of the fever three days postop, and a stable condition was achieved 12 days postop [1]. No recurrence of XPG in the contra-lateral kidney has yet been reported [13].

## Conclusions

Xanthogranulomatous pyelonephritis is a rare and severe disease that can result in fatal complications if not diagnosed and treated early. It can mimic other neoplastic disorders and inflammatory processes, which makes diagnosis challenging. However, with advancements in imaging and radiological technology, a preoperative diagnosis can be made early. Treatment by nephrectomy is usually curative, and correct preoperative diagnosis is crucial to minimize the spread of infected fluid into the peritoneum. It is important to consider XGP as a differential diagnosis in young children presenting with symptoms of an abscess, renal mass, or non-functioning kidney with or without chronic renal calculus disease.
